# Marine Toxin Okadaic Acid Affects the Immune Function of Bay Scallop (*Argopecten irradians*)

**DOI:** 10.3390/molecules21091108

**Published:** 2016-08-24

**Authors:** Cheng Chi, Sib Sankar Giri, Jin Woo Jun, Hyoun Joong Kim, Saekil Yun, Sang Guen Kim, Se Chang Park

**Affiliations:** Laboratory of Aquatic Biomedicine, College of Veterinary Medicine and Research Institute for Veterinary Science, Seoul National University, Seoul 151742, Korea; chicheng0421@126.com (C.C.); giribiotek@gmail.com (S.S.G.); advancewoo@hanmail.net (J.W.J.); hjoong1@nate.com (H.J.K.); arseidon@naver.com (S.Y.); imagine5180@gmail.com (S.G.K.)

**Keywords:** okadaic acid, harmful algal blooms, bay scallop, *Argopecten irradians*, immune response

## Abstract

Okadaic acid (OA) is produced by dinoflagellates during harmful algal blooms and is a diarrhetic shellfish poisoning toxin. This toxin is particularly problematic for bivalves that are cultured for human consumption. This study aimed to reveal the effects of exposure to OA on the immune responses of bay scallop, *Argopecten irradians*. Various immunological parameters were assessed (total hemocyte counts (THC), reactive oxygen species (ROS), malondialdehyde (MDA), glutathione (GSH), lactate dehydrogenase (LDH), and nitric oxide (NO) in the hemolymph of scallops at 3, 6, 12, 24, and 48 h post-exposure (hpe) to different concentrations of OA (50, 100, and 500 nM). Moreover, the expression of immune-system-related genes (*CLT-6*, *FREP*, *HSP90*, *MT*, and *Cu*/*ZnSOD*) was also measured. Results showed that ROS, MDA, and NO levels and LDH activity were enhanced after exposure to different concentrations of OA; however, both THC and GSH decreased between 24–48 hpe. The expression of immune-system-related genes was also assessed at different time points during the exposure period. Overall, our results suggest that exposure to OA had negative effects on immune system function, increased oxygenic stress, and disrupted metabolism of bay scallops.

## 1. Introduction

Harmful algal blooms (HABs), caused by pollution of water bodies and global climate change, can result in ecological and economic losses in coastal areas [1]. HABs also have various negative impacts on public health and threaten the aquaculture industry [2], as they cause mass mortality of cultivated animals from the algal toxins they produce [1]. The main marine phycotoxins which can be ultimately consumed by humans, causing a variety of gastrointestinal and neurological illnesses through the food chain are shellfish toxins, including paralytic shellfish poisoning (PSP), diarrhetic shellfish poisoning (DSP), amnesic shellfish poisoning (ASP), neurotoxic shellfish poisoning (NSP), and azaspiracid shellfish poisoning (AZP) [3]. The DSP toxins include okadaic acid (OA), the dinophysistoxins-1 (DTX-1), DTX22, DTX-3, and their derivative forms [4]. These are produced by some microalgae of the genera *Dinophysis* and *Prorocentrum,* such as *P. lima*, *P. concavum*, *P. maculosum*, *D. acuminata*, *P. rhathymum*, and *D. fortii* etc. [3]. These toxins can accumulate in the fatty tissue of bivalves [4]. Among them, OA and its derivatives are the best representative of DSP toxins [5], which inhibit serine-threonine protein phosphatase 1 and phosphatase 2A, leading to metabolic process deregulation and hyperphosphorylation of many cellular proteins [6]. Previous studies have reported that exposure to OA or *P. lima* cause damage to hemocyte function and the viability of carpet shell clams (*Ruditapes decussatus*) [6]. Huang et al. [3] also revealed that *P. lima*, a dinoflagellate producing OA, induced cytoskeleton disorganization, oxidative stress, and dysfunction of metabolism in mussels.

Bivalves are particularly affected during HAB events as they accumulate high levels of algal toxins in their tissues through their sessile and filter-feeding habits [1]. Therefore, previous studies of the impact of algal toxins have focused on oysters, mussels, or clams shells following exposure to toxic algae [1,3,6,7,8,9,10,11]. To date, there is little data on the effects of algal toxins on scallops. Moreover, most of these studies investigated the effects of phycotoxins by feeding or exposing mussels and oysters to harmful algae, which secrete various phycotoxins and other metabolites. However, the direct impacts of purified toxins on physiological responses in scallops have rarely been investigated [3,7,8,9,10,11].

Scallops are a cosmopolitan family of bivalves, some of which are widely farmed by the aquaculture industry for food and have important economic value. The bay scallop (*Argopecten irradians*) was introduced to China from America and has been cultured in the coastal provinces of China for more than 30 years, and now bay scallop farming is also suffering from HABs [12]. Scallops accumulate toxins in their tissues to a greater extent as they have a low metabolic rate [13]. Hemocytes are important in the immune responses of bivalves as they are involved in the inflammatory response, respiratory burst, wound recovery, phagocytosis, and encapsulation [14]. During phagocytosis, large amounts of reactive oxygen species (ROS) are generated to kill the internalized bacteria, which is important for invertebrate survival [12]; however, damage occurs as a result of excessive generation of ROS [13]. Excessive production of ROS and other pro-oxidants damage unsaturated lipids, and break DNA bonds, proteins, amino acids, and carbohydrates [14]. In scallops, superoxide dismutase (SOD) is considered as the first and most important line of defense against ROS and protects tissues from oxidative damage [15]. Glutathione (GSH) is another well-known antioxidant defense. Both GSH and SOD are frequently used as biomarkers in aquatic species, including scallops [15,16]. GSH can directly neutralize several reactive species by being oxidation to oxidized glutathione (GSSG), and also acts as a cofactor of several antioxidant glutathione-dependent enzymes. Lactate dehydrogenase (LDH) is a soluble cytosolic enzyme and is widely used as a biomarker in toxicology and chemical threats to evaluate the status of cell, tissue, and organ damage [17]. Any changes in the level of LDH activity suggest metabolic changes in the affected tissues [18]. Nitric oxide (NO) is a crucial gaseous signaling molecule that is involved in a series of disease pathogenesis and physiological processes in invertebrates, including immune defense [19]. However, NO can also react indirectly with ROS to produce a more powerful oxidant peroxynitrite, which prevents DNA repair and is closely related to apoptosis [19]. In addition, malondialdehyde (MDA) levels represent membrane lipid peroxidation status and are also used as a marker to determine the extent of oxidative damage [14].

The multiple factors and complexity of feeding in the effects of exposure to toxic microalgae, especially during natural HAB outbreaks, are particularly problematic for the safe and efficient culturing of bivalves, such as scallops, for human consumption. This study evaluated the effect of OA on scallops to gain a better understanding of the toxicity of DSP toxins and help improve the intensive breeding and long-term sustainability of scallop farming [12]. We compared the immunotoxicity parameters (THC, ROS, MDA, NO, GSH, and LDH) in the hemolymph of bay scallops following exposure to different concentrations of OA to understand the early physiological and immunological responses of bay scallops to the toxicity of DSP toxins and provide information on its molecular mechanism of the responses of bay scallops. In addition, we examined the transcription levels of several immune-system-related genes (*CLT-6*, *FREP*, *HSP90*, *MT*, and *Cu/ZnSOD*). To our knowledge, this is the first study comparing the effect of purified OA on bay scallop physiological and immunological responses and the expression of immune-system-related genes.

## 2. Results

### 2.1. Non-Specific Immune Responses

#### 2.1.1. Total Hemocyte Count (THC)

**Figure 1:**
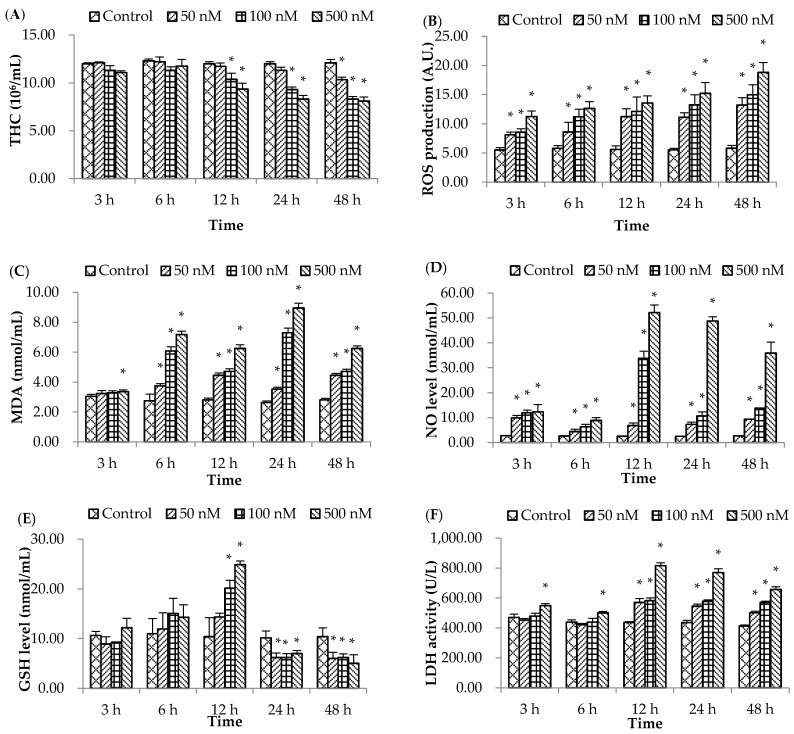
Effects of okadaic acid (OA) on non-specific immune responses of the bay scallop *Argopecten irradians* at different time intervals after exposure to three concentrations (50, 100, and 500 nM) of OA. (**A**) total hemocyte counts (THC); (**B**) reactive oxygen species (ROS); (**C**) malondialdehyde (MDA); (**D**) nitrite oxide (NO) level; (**E**) glutathione (GSH) level; and (**F**) lactate dehydrogenase (LDH) activity. Data represent mean ±SD values (*n* = 9) at the same time interval with different letters denoting significant differences (*p* < 0.05).

The THC (Figure 1A) decreased in groups exposed to 100–500 nM of OA for 12–48 h post-exposure (hpe) (*p* < 0.05). However, significant reduction of THC was also observed in the group exposed to 50 nM OA at 48 hpe. 

#### 2.1.2. Reactive Oxygen Species (ROS) Level

The ROS level (Figure 1B) was increased (*p* < 0.05) at all time intervals following exposure to three concentrations of OA, and gradually increased with increasing exposure time.

#### 2.1.3. Malondialdehyde (MDA) Level

The level of MDA was higher (*p* < 0.05) in all of the OA treated groups at 6–48 hpe (Figure 1C) with a significant increase at 3 hpe at 500 nM of OA. In each time interval, the highest level of MDA was observed at 500 nM of OA, followed by exposure to 100 nM, and 50 nM of OA. 

#### 2.1.4. Nitric Oxide (NO) Level

The level of NO was significantly increased in all of the OA-treated groups at all time intervals (Figure 1D) with the highest level at 12 hpe at 100–500 nM of OA. 

#### 2.1.5. Glutathione (GSH) Level

The level of GSH (Figure 1E) showed no significant changes in any of the OA-treated groups at 3–6 hpe; however, a higher GSH level in 100–500 nM OA-treated groups was observed at 12 hpe. Thereafter, the GSH level in all of the OA-treated groups sharply decreased (*p* < 0.05) at 24–48 hpe.

#### 2.1.6. Lactate Dehydrogenase (LDH) Activity

The LDH activity (Figure 1F) was greater (*p* < 0.05) in the 500 nM OA treatment group at all time intervals compared to the control group; however, significant increments (*p* < 0.05) of LDH activities in scallops treated with 50 and 100 nM of OA were observed at 12–48 hpe.

### 2.2. Expression of Immune-System-Related Genes

#### 2.2.1. Expression of *CTL-6* Gene

**Figure 2:**
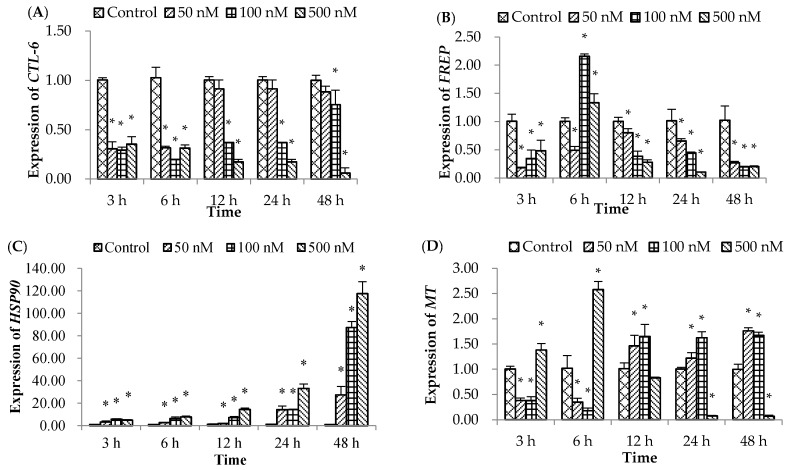

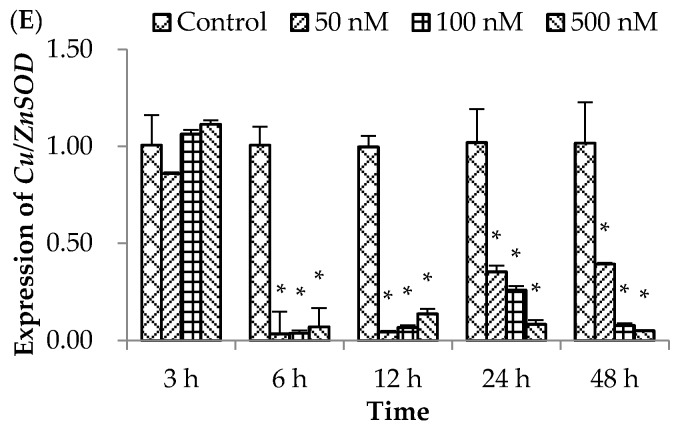
Effects of okadaic acid (OA) on non-specific immune responses in the bay scallop *Argopecten irradians* at different time intervals following exposure to three concentrations (50, 100, and 500 nM) of OA. (**A**) *CTL-6* gene; (**B**) *FREP* gene; (**C**) *HSP90* gene; (**D**) *MT* gene; and (**E**) *Cu*/*ZnSOD* gene. Data represent mean ± SD values (*n* = 9) at the same time intervals with different letters denoting significant differences (*p* < 0.05).

*CTL-6* gene expression (Figure 2A) in the three OA treatment groups was lower (*p* < 0.05) than that in the control group at 3–48 hpe, except in the 50 nM OA treatment group at 12–48 hpe, which returned to normal levels compared to the control.

#### 2.2.2. Expression of *FREP* Gene

*FREP* gene expression (Figure 2B) in the 50 nM of OA treatment group was down-regulated (*p* < 0.05) at 3–48 hpe. However, *FREP* gene expression in the 100 and 500 nM of OA treatment groups significantly decreased at 3 hpe, and sharply increased at 6 hpe, and thereafter sharply decreased from 12–48 hpe.

#### 2.2.3. Expression of *HSP90* Gene

The expression of *HSP90* mRNA (Figure 2C) in the hemolymph treated with three concentrations of OA gradually increased (*p* < 0.05) up to 48 h with increasing exposure time, reaching the highest levels at 48 hpe.

#### 2.2.4. Expression of *MT* Gene

Scallops treated with 50 and 100 nM of OA had lower (*p* < 0.05) *MT* expression from 3–6 hpe, but higher (*p* < 0.05) *MT* expression at 12–48 hpe (Figure 2D). Conversely, *MT* expression was strongly induced at 3–6 hpe in the 500 nM of OA group, and then returned to a normal (control) level at 12 hpe, thereafter, it was obviously suppressed at 24–48 hpe.

#### 2.2.5. Expression of *Cu*/*ZnSOD* Gene

Expression of the *Cu*/*ZnSOD* gene (Figure 2E) in the hemolymph treated with the three concentrations of OA showed no significant changes at 3 hpe; however, it was continuously down-regulated (*p* < 0.05) from 6–48 hpe, compared to the control group.

## 3. Discussion

Okadaic acid (OA) is the main marine toxin responsible for the DSP that causes gastrointestinal symptoms in humans following consumption of contaminated bivalves. OA, a potent and non-selective inhibitor of serine/threonine phosphatases, has been shown to be cytotoxic in a variety of cell lines [20]. Previous reports revealed that constant contact with OA induced chromosome loss, apoptosis, DNA damage, and inhibited phosphatases in contaminated bivalves. In this study, immune-system responses and expression of immune-system-related genes were determined in the hemolymph of bay scallops following exposure to different concentrations of OA.

Several immune parameters have been successfully employed to evaluate the immune status of bivalves subjected to various stresses [21]. The total hemocyte count (THC) is one of the most widely used parameters to assess bivalve health status as the number of circulating hemocyte changes under stressful conditions [9]. In this study, THC decreased significantly in the medium to high concentrations of OA-treatment groups after 12 hpe. A similar situation was observed in the clam, *Ruditapes philippinarum*, where the THC decreased upon exposure to the dinoflagellate *Prorocentrum minimum*, which produces algal diarrheic shellfish toxins, such as OA and dinophysistoxins [9,22]. Exposure to higher concentrations of OA for longer periods resulted in higher apoptosis. This suggests that OA induced hemocyte death in a dose and time-dependent manner. Similarly, previous reports have revealed that OA induced apoptosis even in human monocytic U-937 cells [20]. Therefore, this result suggests that OA exposure could disturb and restrain the immune response of bay scallops. ROS production is another important mechanism of bivalve cellular immunity. Although a small amount of ROS is necessary to enhance the internal defense against pathogens, serious damage to lipids, proteins, and DNA occurs when the generation of ROS is excessive [13]. A significant increase in ROS in OA-treated scallops was observed during all time intervals, and ROS generation was time and dose-dependent. This is in agreement with the previous report of OA-induced ROS generation in human monocytic U-937 cells [20]. Therefore, our results indicated that exposure to OA generated ROS in scallops.

Malondialdehyde (MDA) is closely related to the membrane lipid peroxidation status; therefore MDA content assay is used to indirectly evaluate the extent of oxidative damage [23]. In this study, exposure to OA resulted in significant increases in MDA content in all time intervals, which continued to increase as the exposure time increased. The current results are agreement with previous study, which reported that MDA level in the hemolymph of the temperate scallop *Pecten maximus* significantly increases with exposure to phenanthrene [24].This phenomenon indicated that the hemolymph suffered from serious oxidative stress and oxidative damage to macromolecules. In addition, the NO level was significantly increased following exposure to OA, and sharply increased from 12 hpe in the group exposed to highest concentration of OA. In bivalves, NO is an essential molecule that is related to normal physiological functions [25], such as the regulation of neural transmission, vascular tone, and immune defense [19]. Although NO is not toxic in itself, during phagocytosis, in combination with superoxide anions synthesis, it generates the highly toxic peroxynitrite anion (ONOO-) [25]. Recently, it has been shown that production of the physiological messenger NO increased after treatment with the polyunsaturated aldehyde decadienal produced by diatoms in embryos of the sea urchin *Paracentrotus lividus* [26]. Moreover, a significant increase in NO production was reported in sea urchin embryos and in the different developmental stages of the offspring derived from females of the sea urchin exposed to heavy metal (e.g., cadmium and manganese ) contamination [27,28]. C.M. de Barros et al. [29] also reported that zymosan A and lipopolysaccharide (LPS) enhanced NO production in hemocytes of the ascidian *Phallusia nigra*. Our results of the present investigation are in accordance with the report of Migliaccio et al. [30] that adult sea urchin showed high nitric oxide (NO) levels, along with a low fertilization rate after toxic blooms of *Ostreospsis cf. ovata*. These previous studies indicated that Zymosan A, LPS, or other soluble or particulate non-self materials could stimulate NO production in mollusk. Therefore, the results of our study suggests that NO mediates the stress response of bay scallop against the toxic effects of OA. Lactate dehydrogenase (LDH) is a soluble cytosolic enzyme present in most living cells. This enzyme catalyzes the reversible oxidation of l-lactate to pyruvate, with nicotinamide adenine dinucleotide (NAD+) as a hydrogen acceptor in the final step of the metabolic chain of anaerobic glycolysis [17,31]. Therefore, LDH is widely used as a biomarker in toxicology and clinical chemistry to diagnose cell, tissue, and organ damage. In this study, bay scallops treated with different concentrations of OA produced notable increases in LDH activity in the hemolymph. This increase in LDH activity reflected OA damage to tissues or apoptosis. Ravindran et al. [20] also reported a significant increase in LDH activity in U-937 cells from OA exposure after 4 h, reaching a maximum at 16 h. Traoré et al. [32] reported that 15 ng/mL of OA increased the release of LDH in Vero cells.

Pollutants can produce free radical O_2_ and H_2_O_2_ through biotransformation, both of which may cause oxidative stress if not metabolized quickly [33]. Glutathione (GSH), which catalyzes H_2_O_2_ to molecular water, plays a crucial role in protecting organisms from oxidative stress. Although a significant increase in GSH level was observed in the OA treatment groups at 12 hpe, which reflects an up-regulation of antioxidant defenses and, thereafter, it gradually decreased in all treatment groups, may be due to overwhelming of the antioxidant capacity that can lead to mass oxidation of GSH resulting in excretion of the oxidised molecule (GSSG) from the cell leading to a reduced intracellular concentration of GSH level [34]. Ravindran et al. [20] also reported a significant decrease in GSH in OA-treated U-937 cells. Moreover, Zhang et al. [35] revealed that GSH levels in one-month-old mice reduced at 24 h after intraperitoneal injection of OA. In addition, a similar reduction in total glutathione has been reported in scallop exposed to the acute oil after 48 h [34]. Superoxide dismutase (SOD) is another important antioxidant enzyme that catalyzes conversion of superoxide to oxygen and H_2_O_2_, and has been considered as a suitable indicator for ecological risk assessments [32]. Based on the metal ion cofactor in the active site, SODs are classified into iron SOD (Fe SOD), manganese SOD (Mn SOD), nickel SOD, and copper-zinc SOD (Cu, Zn SOD); the Cu/ZnSODs have two forms: cytosol dimmers and extracellular tetramers [36]. In the hemolymph of bay scallops, the expression of the *Cu*/*ZnSOD* gene was significantly down-regulated in all of the OA-treated groups at 6–48 hpe. This indicated that OA inhibited the expression of the *Cu*/*ZnSOD* gene, and inhibited the antioxidant abilities of scallops exposed to OA. Similarly, Zhang et al. [35] revealed that SOD activity in one-month-old mice was significant lower than that in the control group at 24 h after intraperitoneal injection of OA, similar to our results. Results of the present study suggest stimulated ROS production exceeded the neutralizing capabilities of the antioxidant system and reduced the GSH level.

In addition, heat shock proteins (HSPs) play a significant role in preventing irreversible protein denaturation, promoting either repair or destruction of damaged proteins. The expression of the *HSP90* gene notably increased after exposure to OA in a dose and time-dependent manner, suggesting that HSPs (*HSP90* gene) protected the bay scallop against higher concentrations of OA as the exposure time increased. These results are consistent with those reported by Manfrin et al. [37] who showed that the expression of the *HSP90* gene in the mussel *Mytilus galloprovincialis* was significantly induced by OA exposure. Wang et al. [21] also reported that expression of the *HSP90* gene was significantly induced after 24 h exposure to environmental ammonia-N. Although OA exposure stimulated the antioxidant systems of scallops, significant increases in MDA, ROS level, and LDH activity in the hemolymph of scallops were observed. This reflected the limited abilities of antioxidant systems in scallops to fully remove these harmful superoxide radicals, resulting in oxidative damage to macromolecules.

Metallothionein (MT) is a group of molecules involved in responses to oxidative stress, especially from toxic metals [25]. MT induction has also been found to respond to tissue injury, infection, and inflammation; therefore, MT is probably important in the immune system of scallops [38,39]. In our study, expression of the *MT* gene was significantly suppressed in the 50 and 100 nM OA-treated groups up to 6 hpe, and then markedly increased until 48 hpe; however, the result was opposite in the highest concentration of OA treated group. A previous study also revealed [23] that expression of the *MT* gene was up regulated by 80 mg/L of a chemical contaminant palmitoleic acid (PA) in a short-space of time at 12 hpe, and was suppressed up to 48 hpe. This phenomenon may be attributed to the high concentration of OA modulating *MT* gene expression in a short exposure period and inducing *MT* mRNA transcription to briefly counter the tissue injury or oxidative stress, but finally strongly inhibiting expression. However, the lower concentrations took more time to modulate *MT* gene expression, and it could be that the *MT* mRNA transcription was inhibited with the increasing exposure time. In general, these results reflect the weakened ability of scallops treated with OA to respond to oxidative stress, tissue injury, infection, and inflammation.

C-type lectins act as a first line of defense against pathogens; they recognize and bind to terminal sugars on glycoproteins and glycolipids, and play significant roles in non-self recognition and the clearance of foreign particles, either as cell surface receptors for microbial carbohydrates or as soluble proteins existing in scallop tissue fluids [37]. We found that scallops exposed to the lowest concentration of OA attenuated the expected expression of *CLT-6* mRNA at 3–6 hpe, with the higher concentrations of OA producing significantly higher *CLT-6* expression at each time interval. These results are consistent with the previous investigation in which PA exposure modulated the expected expression of the *CLT-6* gene, suggesting that the antibacterial or antivirus ability of scallops was negatively impacted by OA exposure [23]. Fibrinogen-related protein (FREP) is another pattern recognition receptor in innate immune system responses, especially in the clearance of non-self materials, such as microorganisms and late apoptotic cells through the lectin pathway [40]. In this study, expression of the *FREP* gene was inhibited in all of the treatment groups at 3–48 hpe, except 100 and 500 nM at 6 hpe. These results indicated that OA exposure inhibited *CLT-6* and *FREP* gene expression, and then restrained the ability of scallops to recognize and clear non-self particles, such as microorganisms and late apoptotic cells through the lectin pathway. 

In conclusion, this study shows that the bay scallop, *A. irradians* was affected immunologically by OA exposure. Altogether, these results showed significant changes in several of the immune-system-related parameters that we monitored (THC, ROS, MDA, LDH, NO, and GSH) indicating that the toxin OA can slowly diminish the immune system of bay scallop, especially when they are exposed to high concentrations of toxins or a long exposure period. Furthermore, significant changes in expression of immune response related genes (*CLT-6*, *FREP*, *HSP90*, *Cu*/*ZnSOD*, and *MT*) suggests cellular stresses. This study revealed OA-induced immunological and physiological effects on a species of bivalve—the bay scallop—and helps to establish the species of bivalves that are immunologically more sensitive to algal toxins. This could serve to better control potential infections, inflammation, or other oxidative damage that may further affect cultivated scallops already immuno-depleted by the algal toxin OA.

## 4. Materials and Methods 

### 4.1. Okadaic Acid

Okadaic acid (OA), 92%–100% (HPLC), was obtained from Sigma-Aldrich Co. LLC (Sigma, St. Louis, MO, USA) and stored at 4 °C in a refrigerator until use.

### 4.2. Animals

Bay scallops, *A. irradians*, averaging 60–70 mm in shell length, were collected from the Noryangjin fisheries wholesale market (Seoul, Korea) and maintained in lantern nets suspended in 800-L-capacity tanks containing filtered and aerated sea water to acclimatize to laboratory conditions (temperature: 10 ± 1 °C; salinity: 30‰ ± 0.1‰) for two weeks. Half of the seawater was changed every day. Scallops were fed a commercial shellfish diet (Instant Algae^®^ Shellfish Diet; Reed Mariculture Inc., Campbell, CA, USA) at a rate of approximately 1.2 × 10^10^ algae cells per scallop per day. 

Three hundred and sixty bay scallops (mean 46.02 ± 2.67 g) were randomly divided into a control (without OA) and treatment groups (with OA). Each group consisted of 30 scallops with three replicates (30 × 3 = 90 per group) The OA treatment groups were treated with one of three concentrations (50, 100, and 500 nM) of OA. The three OA concentrations were selected based on previous studies reporting cytotoxic and genotoxic effects of OA on different cell lines [6]. Three scallops from each replicate treatment group were randomly collected at 3, 6, 12, 24, and 48 h after exposure to OA. Two mL of hemolymph were collected from each adductor muscle using a 1-mL sterile syringe fitted with a 22-gauge needle within 1 min of removing a scallop from the tank. Individual scallops were sampled once to avoid repeatedly drawing blood and/or handling stress. A 100-µL sample of hemolymph from each replicate treatment group was used for RNA extraction. A 20-µL sample of hemolymph was diluted 1:3 with Baker’s Formol Calcium (2% sodium chloride, 1% calcium acetate, 4% formaldehyde) to fix cells and prevent aggregation for total hemocyte count [13]. The remaining hemolymph from each replicate treatment group was centrifuged at 750× *g* for 3 min to collect the serum, which was then stored at −80 °C until testing for humoral immune parameters. 

### 4.3. Measurement of Non-Specific Immune Responses

#### 4.3.1. Total Hemocyte Count

A sample of 100 μL hemolymph was first fully mixed with an equal volume of Tris-EDTA (18 mM Tris; 0.45 M NaCl; 13 mM KCl; 16 mM d-glucose; 20 mM EDTA; pH 7.5) to avoid haemocyte agglutination, and then added to a hemacytometer, and then total hemocyte count (THC) was calculated as cells per mL using an improved Neubauer hemocytometer under 40× magnification [41]. Counts were performed in triplicate and the mean and standard deviation calculated.

#### 4.3.2. Measurement of Reactive Oxygen Species Production

Reactive oxygen species (ROS) production was measured using reactive oxygen species kits (Nanjing Jiancheng Bioengineering Institute, Nanjing, China) following the manufacturer instructions. Fluorescence, quantitatively related to the ROS production of hemocytes without any stimulation, was measured at 500–530 nm by a fluorescence microplate reader. Fluorescence was expressed in arbitrary units (A.U.).

#### 4.3.3. Measurement of Malondialdehyde Content

Malondialdehyde (MDA), a degradation product of lipid peroxidation known as thiobarbituric acid-reactive substance, was determined according to the thiobarbituric acid method using a MDA test kit and following the manufacturer’s instructions (Nanjing Jiancheng Bioengineering Institute). The MDA in decomposed products of lipid peroxidation can have condensation reaction with thio-barbituric acid, generates a red product which has a maximum absorption peak at 532 nm (wavelength).

#### 4.3.4. Measurement of Nitric Oxide Assay

Nitrite oxide (NO) level was estimated enzymatically using a commercial test kit (Nanjing Jiancheng Bioengineering Institute) according to the manufacturer’s instructions. NO is oxidized by oxygen to nitrate and nitrite which reacts with chromogenic agent to generate azo dyes and can be measured spectrophotometrically.

#### 4.3.5. Measurement of Glutathione Assay 

The content of reduced glutathione (GSH) in hemolymph was measured with an assay kit (Nanjing Jiancheng Bioengineering Institute). DTNB (5,5′-Dithiobis(2-nitrobenzoic acid)) was developed for the detection of thiol compounds. DTNB and glutatuhione react to generate 2-nitro-5-thiobenzoic acid and glutathione disulfide (GSSG). Since 2-nitro-5-thiobenzoic acid is a yellow colored product, GSH concentration in a sample solution can be determined by the measurement at 420 nm absorbance.

#### 4.3.6. Measurement of Lactate Dehydrogenase Assay

Lactate dehydrogenase (LDH) released from hemolymph was measured using a lactate dehydrogenase assay kit (Nanjing Jiancheng Bioengineering Institute) according to the manufacture instructions. LDH can catalyze lactic acid to pyruvic acid, pyruvic acid reacts with 2,4-dinitrophenylhydrazine to produce pyruvic dinitrophenylhydrazone. The product appears red-brown in alkaline solution, and enzyme activity can be determined by measuring absorbance.

### 4.4. RNA Extraction and Reverse Transcription

Total RNA was extracted from hemolymph using TRIzol Reagent (CWBio, Beijing, China). The quality and purity of RNA was assessed by spectrophotometry, and the 260:280 ratios were 1.8:2.0. Genomic DNA contamination was removed using DNase I (Promega, Madison, WI, USA). cDNA was synthesized using a PrimeScript^TM^ RT Reagent Kit (TaKaRa Bio, Shiga, Japan) following the manufacturer’s instructions. The cDNA so obtained was stored at −80 °C.

### 4.5. Real-Time Quantitative PCR Analyses of Gene Expression

The expression of immune-system-related genes *CLT-6*, *FREP*, *HSP90*, *MT*, and *Cu*/*ZnSOD* was performed using real-time quantitative PCR (qPCR) (Qiagen, Hilden, Germany). All qPCR reactions were performed using SYBR Premix Ex Taq™ Perfect Real-Time Kits (TaKaRa Bio) and were conducted using a Qiagen Rotor-Gene Q RT-PCR Detection System (Qiagen, Hilden, Germany). Gene expression was normalized using the housekeeping gene *β-actin*. PCR primer sequences used for qPCR are listed in Table 1. The reaction mixture included 10 μL SYBR Premix Ex Taq™, 1 μL of the forward and reverse primer (10 mM), and 1 μL cDNA. The remaining volume was filled with ultra-pure water to a final total volume of 20 μL. The reaction conditions and cycle index were conducted at 95 °C for 10 min, followed by 40 cycles at 95 °C for 45 s, 56 °C for 45 s, and 72 °C for 30 s. After the amplification phase, a melting curve analysis was conducted to eliminate the possibility of non-specific amplification or primer dimer formation. A standard curve was created based on serial dilutions of sample cDNA. A standard curve was drawn by plotting the natural log of the threshold cycle (Ct) against the number of molecules. Standard curves for each gene were run in duplicate and triplicate to obtain reliable amplification efficiency. The correlation coefficients (R^2^) of all standard curves was >0.99 and the amplification efficiency was between 90% and 110%. The relative expression ratios of the target genes in the treatment groups versus the control group were calculated according to the following formula: Fold changes = 2^−^^△△^Ct, where △△Ct = [Ct (treatment group) − Ct (treatment *β-actin*)] − [Ct (control group) − (control *β-actin*)] [42]. In all cases, each PCR was carried out with three replicates.

### 4.6. Statistical Analysis

Normality and homogeneity of variance were tested utilizing Kolmogrov–Smirnov and Cochran’s tests, respectively. All percentage data were arcsine-transformed, and the data were analysed with one-way ANOVA. Values are expressed as the arithmetic mean ± standard deviation (SD). Differences were determined using the LSD test in SPSS version 19.0 (IBM Corp., Armonk, NY, USA) with *p*-values < 0.05 indicating statistical significance.

## Figures and Tables

**Table 1:** Primers used for the analysis of mRNA expression by qRT-PCR.

Genes	Primer Sequence	Accession No.
*β-actin*	F: 5′CAAACAGCAGCCTCCTCGTCA 3′	AY335441
	R: 5′CTGGGCACCTGAACCTTTCGTT 3′	
*CTL-6*	F: 5′CAGTTGCTACAGGGTTCG 3′	GQ202279
	R: 5′GGGCGTTATCTGGCTCAT 3′	
*FREP*	F: 5′CGTCGCAAATGCTGAAGATG 3′	EU399719
	R: 5′TAAGTTGTGGTCGGTCCTGAGA 3′	
*HSP90*	F: 5′TCAGTATGGTTGGTCCGCTAA 3′	EF532406
	R: 5′CGGTTGCCTTTTCCTTCAGA 3′	
*MT*	F: 5′AACTTGCTGTAGTGGGAATG 3′	EU734181
	R: 5′AGGCTGGAAACTGCTGTGGT 3′	
*Cu*/*ZnSOD*	F: 5′GTATTGAAAGGTGATTCGGAGG 3′	EU563958
	R: 5′ATGCACATGAAAGCCATGTAGG 3′	
